# Reference values for intracranial pressure and lumbar cerebrospinal fluid pressure: a systematic review

**DOI:** 10.1186/s12987-021-00253-4

**Published:** 2021-04-13

**Authors:** Nicolas Hernandez Norager, Markus Harboe Olsen, Sarah Hornshoej Pedersen, Casper Schwartz Riedel, Marek Czosnyka, Marianne Juhler

**Affiliations:** 1 Department of Neurosurgery, Rigshospitalet, Copenhagen, Denmark; 2 Department of Neuroanaesthesiology, Rigshospitalet, Copenhagen, Denmark; 3 Department of Neurophysiology, Rigshospitalet, Glostrup, Denmark; 4 Brain Physics Laboratory, Department of Clinical Neurosciences, Addenbrooke’s Hospital, University of Cambridge, Cambridge, UK; 5 Department of Neurosurgery, Aarhus University Hospital, Aarhus, Denmark

**Keywords:** Cerebrospinal fluid pressure, CSF, ICP, Intracranial pressure, Reference intervals, Reference values

## Abstract

**Background:**

Although widely used in the evaluation of the diseased, normal intracranial pressure and lumbar cerebrospinal fluid pressure remain sparsely documented. Intracranial pressure is different from lumbar cerebrospinal fluid pressure. In addition, intracranial pressure differs considerably according to the body position of the patient. Despite this, the current reference values do not distinguish between intracranial and lumbar cerebrospinal fluid pressures, and body position-dependent reference values do not exist. In this study, we aim to establish these reference values.

**Method:**

A systematic search was conducted in MEDLINE, EMBASE, CENTRAL, and Web of Sciences. Methodological quality was assessed using an amended version of the Joanna Briggs Quality Appraisal Checklist. Intracranial pressure and lumbar cerebrospinal fluid pressure were independently evaluated and subdivided into body positions. Quantitative data were presented with mean ± SD, and 90% reference intervals.

**Results:**

Thirty-six studies were included. Nine studies reported values for intracranial pressure, while 27 reported values for the lumbar cerebrospinal fluid pressure. Reference values for intracranial pressure were −  5.9 to 8.3 mmHg in the upright position and 0.9 to 16.3 mmHg in the supine position. Reference values for lumbar cerebrospinal fluid pressure were 7.2 to 16.8 mmHg and 5.7 to 15.5 mmHg in the lateral recumbent position and supine position, respectively.

**Conclusions:**

This systematic review is the first to provide position-dependent reference values for intracranial pressure and lumbar cerebrospinal fluid pressure. Clinically applicable reference values for normal lumbar cerebrospinal fluid pressure were established, and are in accordance with previously used reference values. For intracranial pressure, this study strongly emphasizes the scarcity of normal pressure measures, and highlights the need for further research on the matter.

**Supplementary Information:**

The online version contains supplementary material available at 10.1186/s12987-021-00253-4.

## Background

Measurements and analysis of intracranial pressure (ICP) are used to provide information for treating and managing patients with numerous neurological diseases, e.g., traumatic brain injury, acute intracranial hemorrhages, idiopathic intracranial hypertension, and hydrocephalus [1–4]. However, values for normal ICP measured intracranially remain sparsely documented, and the currently used reference interval (7 to 15 mmHg [5]) is based on studies in which lumbar cerebrospinal fluid opening pressure (LCSF_op_) is used as a surrogate parameter for ICP or is extrapolated from patients with a suspected ICP disorder [5–10].

In the 1920s and 30 s, it was assumed that ICP measured intracranially was equal to LCSF_op_ measured at a lumbar spine level [11, 12]. This has, in recent years, been subject to much debate [13–17], and where some studies have documented similar values for intracranial measurements and lumbar measurements [13, 15], others found considerable differences between the two measurement-sites [14, 16, 17]. The reason for a difference might be due to ICP being measured in the brain parenchyma, thus not being measured in the same intracranial space as LCSFop. Thus, ICP measured ventricular might similar to LCSFop, although this has yet to be thoroughly investigated. The comparison of these studies is further hampered by differences in study design, particularly dissimilarities in body position during the measurement and the range of included study participants [13–16, 18, 19]. It is well known that ICP changes with body position [8, 18–21], e.g., ICP is lower in the vertical position compared to the horizontal position [8, 19, 21]. Also, ICP increases significantly with the body placed in lateral recumbent position compared to supine position [18, 22–24]. Based on this, different reference values for ICP should be established for different body positions. This systematic review aims to (1) determine reference values for normal ICP and LCSF_op_, and (2) examine how ICP depends on measurement-site and body position.

## Methods

Prior to initiating this systematic review, the protocol was registered at PROSPERO (October 30, 2019, identification code: CRD42019143018). The review has been conducted in accordance with the PRISMA guidelines, and a completed PRISMA checklist is available in Additional file 1: Appendix 1.

### Eligibility criteria

All included studies had to provide (1) measurements conducted in humans, (2) original values for either ICP or LCSF_op,_ and (3) ICP measured either intracranially (parenchymal, intraventricular, epidural, subdural, or subarachnoid) or LCSF_op_ measured during a lumbar puncture. All age-groups were included. Patients with intracranial pathology that potentially could alter ICP dynamics (e.g., hydrocephalus, idiopathic intracranial hypertension, intracranial hemorrhage, arteriovenous malformation, intracranial tumor, intracranial abscesses or other significant space-occupying processes within the brain) were excluded.

Since measurement of ICP requires invasive measurement methods, the included studies of ICP are based on “pseudo-healthy” patients, which we define as patients requiring neurosurgery for reasons unrelated to ICP, who were considered healthy in terms of ICP and CSF-dynamics.

### Data search

On July 16st 2019, we conducted a search in MEDLINE Ovid (1946 to July 2019), Embase Ovid (1974 to July 2019), Cochrane Central Register of Controlled Trials (CENTRAL) in the Cochrane Library and Science Citation Index Expandex (1900 to July 2019), and Conference Proceeding Citation Index–Science (Web of Science) (1990 to July 2019). There was no restriction on the publication period. We included all peer-reviewed published studies without consideration for publication status or study design. The search was conducted in English, and studies with a non-English title or abstract were thus excluded. Non-English manuscripts, with an English title and abstract, were translated by a native speaker. The search strategy is attached in Additional file 2: Appendix 2. If published data in the included studies were insufficient, the authors were contacted to retrieve raw data. The systematic search was supplemented by a manual reference-search of included studies. Finally, an expert in the field (MJ) was asked to identify any obviously missing studies.

### Study selection and data extraction

Four investigators (CSR, MHO, NHN, SHP) reviewed the studies for eligibility. Two different investigators assessed each study for the title, abstract and full-text screening. Discrepancies were initially resolved between the four investigators, and if this was not possible, an expert (MJ) had the deciding vote. The screening was conducted via Covidence (Covidence systematic review software, Veritas Health Innovation, Melbourne, Australia). Study design, demographics, and information regarding ICP-monitoring were extracted from each study. Study data were extracted from Covidence by NHN and verified by MHO. Disagreements were resolved by discussion.

### Quality assessment

The methodological quality of the studies was independently assessed by two investigators (MHO, NHN). No intervention-based studies were included, and thus, traditional assessment methods for systematic reviews could not be used. Instead, a quality assessment method was created inspired by the Joanna Briggs Institute Critical Appraisal Tool [25]. Two parts of the tool were, however, irrelevant in the assessment of study type and therefore replaced; (1)*“Was the exposure measured in a valid and reliable way?”* was replaced with *“Was the measurement method described in details?”* and (2) *“Were the outcomes measured in a valid and reliable way?”* was replaced with *“Was the included population without current or previous intracranial pathology that could alter ICP dynamics?”*.

### Data synthesis

All statistical analyses were performed in RStudio (R 3.6.2, R Development Core Team (2019), Vienna, Austria). Since all included studies were observational, and the primary purpose of this review was to determine a reference interval for ICP, a conventional meta-analysis was not applicable. Instead, included studies were pooled into two groups, (1) studies in which ICP was measured, and (2) studies in which LCSF_op_ was measured. Raw data were assessed for normality and presented as mean ± standard deviation (SD), 95% confidence intervals (CI), and coherent reference intervals (defined as 5th to 95th percentile) [5]. If statistical values other than SD were presented (e.g., 95% confidence intervals or reference intervals), SD was manually calculated based on the given statistical data according to the Cochrane Handbook of Systematic Reviews [26]. For studies where only the median and interquartile range (IQR) were presented, the median was directly transformed to a mean, while SD was estimated from IQR by SD = IQR/1.35, as recommended in the Cochrane Handbook of Systematic Reviews [26]. Finally, if pressure values were reported in other units (e.g., mmH_2_O or cmH_2_O) it was converted to mmHg.

Normal ICP and LCSF_op_ were subdivided into different body positions stratified by study and presented in a forest plot, including mean values and 95% CI. The corresponding weighted reference intervals for the different body positions were subsequently presented in a table for ICP and LCSF_op_. Pressure measurements are presented in both mmHg and cmH_2_O. The different body positions were compared using a two-tailed Students t-test, and P-values were presented. Due to multiplicity, we chose to Bonferroni-correct in comparison of body positions and thereby lowering the risk of type 1 errors. P-values < 0.05 were considered significant. The included studies provided insufficient data to perform a meaningful multivariate analysis adjusting for age, gender, monitoring equipment, neck position, and zero point for ICP measurement-site.

## Results

**Fig. 1:**
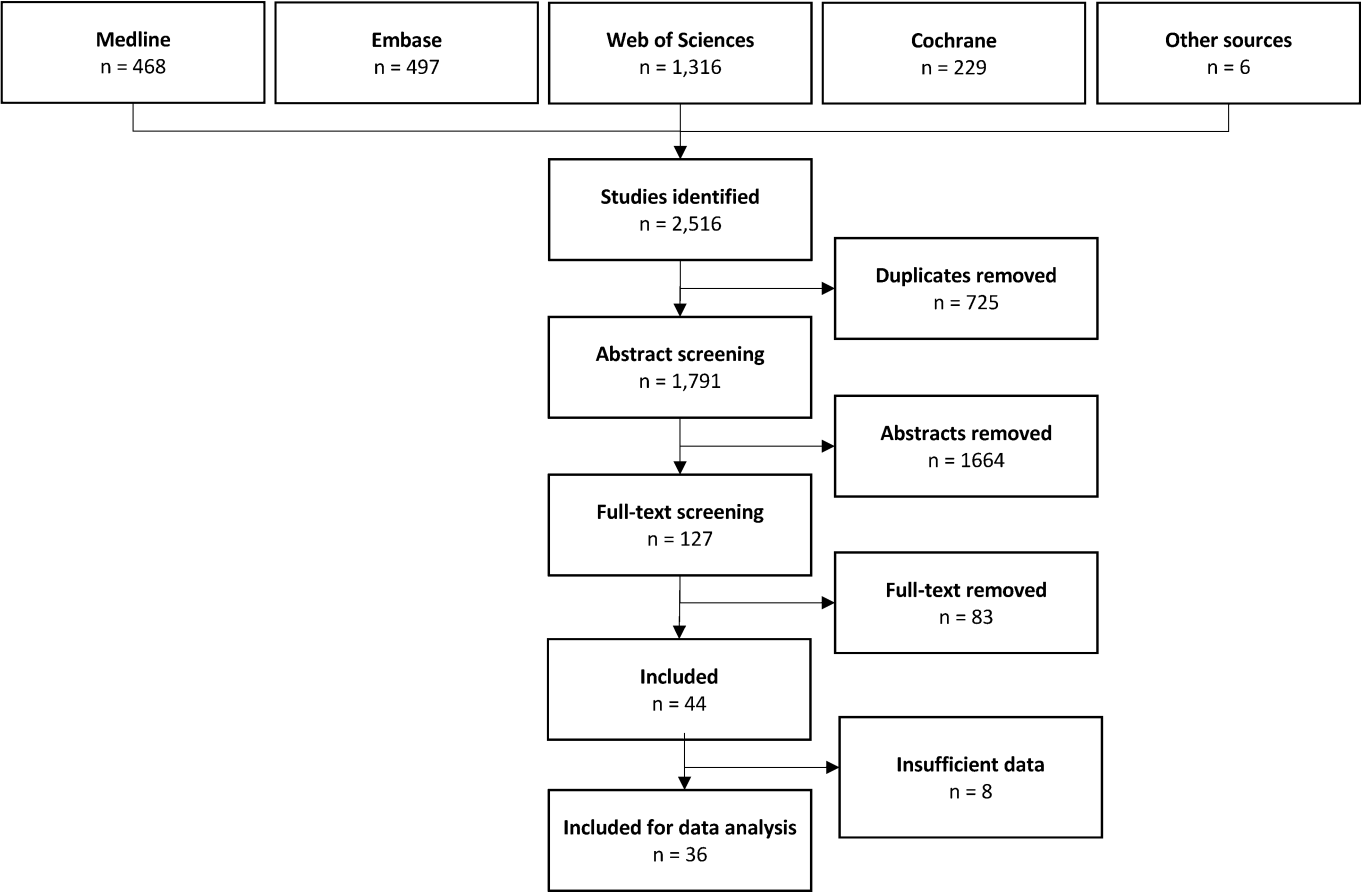
Flowchart of study selection process. The section “insufficient data” covers studies did not report sufficient statistical data to be included in statistical analysis. Thus, no standard deviations, confidence intervals or reference intervals were reported in these studies. The included articles “Other sources” were found by manual searching the reference list of included studies

The search strategy identified a total of 2516 studies. Of these, six were found from other sources than our search string. All these six studies were found by manually searching reference list of included studies. After the removal of duplicates, 1791 studies remained. The abstract and title screening left 127 studies, and based on full-text screening, 44 studies were included (Fig. 1). Study characteristics are presented in Table 1.

**Table 1:** Characteristics of included studies

	Type of study	Type of population	Measurement place	No. of participant	Mean age or range	Included in analysis^a^
Albeck [33]	Prospective, observational	Healthy	Lumbar	8	22–28	Yes
Albeck [34]	Prospective, observational	Healthy	Lumbar	52	60	Yes
Andresen [46]	Prospective, observational	Pseudo-healthy	Parenchymal	4	67	Yes
Avery [63]	Prospective, observational	Pseudo-healthy	Lumbar	197	1–18	Yes
Avery [70]	Case report, literature review	Pseudo-healthy	Lumbar	1	12	Yes
Beck [66]	Prospective, observational	Pseudo-healthy	Lumbar	17	–	Yes
Blomquist [73]	Prospective, observational	Pseudo-healthy	Lumbar	18	0–15	Yes
Bono [67]	Prospective, observational	Pseudo-healthy	Lumbar	111	40	Yes
Bø [69]	Prospective, observational	Pseudo-healthy	Lumbar	348	47	Yes
Chiari [76]	Retrospective, observational	Pseudo-healthy	Parenchymal	41	43	Yes
Chapman [21]	Prospective, observational	Pseudo-healthy	Ventricular	5	28	Yes
Corbett [25]	Prospective, observational	Healthy	Lumbar	15	–	Yes
Eklund [32]	Prospective, observational	Healthy	Lumbar	11	46	Yes
Ekstedt [9]	Prospective, observational	Pseudo-healthy	Lumbar	100	–	Yes
Ellis [24]	Prospective, observational	Pseudo-healthy	Lumbar	33	8	Yes
Fleischman [68]	Retrospective, observational	Pseudo-healthy	Lumbar	12,118	54	Yes
Friden [72]	Retrospective, observational	Pseudo-healthy	Lumbar	150	–	Yes
Gilland [8]	Retrospective, observational	Healthy	Lumbar	15	25	Yes
Gilland [7]	Prospective, observational	Healthy	Lumbar	31	23	Yes
Gonzalez [62]	Prospective, case–control	Pseudo-healthy	Lumbar	28	41	No
Hannerz [35]	Prospective, observational	Healthy	Lumbar	19	52	Yes
Kaiser [60]	Prospective, observational	Pseudo-healthy	Lumbar	49	30 (24–41) weeks	Yes
Kawasaki [61]	Retrospective, case–control	Pseudo-healthy	Lumbar	1	51	Yes
Lakke [36]	Prospective, observational	Healthy	Lumbar	34	–	No
Langvatn [77]	Retrospective, observational	Pseudo-healthy	Parenchymal	12	3	Yes
Lawley [56]	Prospective, observational	Pseudo-healthy	Ventricular	8	35	Yes
Lee [59]	Retrospective, observational	Pseudo-healthy	Lumbar	44	9	Yes
Lundberg [57]	Prospective, case–control	Pseudo-healthy	Ventricular	1	45	No
Magneli [54]	Prospective, case–control	Pseudo-healthy	Parenchymal	1	8	Yes
Mahr [78]	Prospective, observational	Pseudo-healthy	Parenchymal	35	73	No
Malm [5]	Prospective, observational	Healthy	Lumbar	40	70	Yes
Martin [79]	Prospective, case–control	Pseudo-healthy	Intracranial	1	19	No
Pedersen [41]	Retrospective, observational	Pseudo-healthy	Parenchymal	35	4–85	Yes
Petersen [55]	Prospective, observational	Pseudo-healthy	Parenchymal and ventricular	11	44	Yes
Puhringer [37]	Prospective, observational	Healthy	Lumbar	5	25–38	Yes
Purvin [52]	Retrospecitve, case–control	Pseudo-healthy	Lumbar	–	–	No
Riedel [42]	Prospective, observational	Pseudo-healthy	Parenchymal	44	60	Yes
Schwartz [58]	Prospective, observational	Pseudo-healthy	Lumbar	55	56	Yes
Shapiro [29]	Prospective, observational	Healthy	Lumbar	23	0–55	Yes
Skau [30]	Prospective, case–control	Healthy	Lumbar	20	–	Yes
Skipper [31]	Retrospective, case–control	Healthy	Lumbar	24	33	No
Sugita [64]	Prospective, case–control	Pseudo-healthy	Lumbar	3	38	No
Whiteley [11]	Retrospective, observational	Pseudo-healthy	Lumbar	242	–	Yes
Wibroe [65]	Prospective, observational	Pseudo-healthy	Lumbar	28	37	Yes

^a^ Studies were not included in the analysis, if they provided insufficient stastical data to calculate a mean and standard deviation

The majority of studies reported a mean value with coherent SD or a mean value with either 95% CI or reference intervals, from which SD could be calculated (n = 32). In a few studies, only median and IQR were reported (n = 4). Studies that only reported a range or a mean and no further statistical data could be provided from the authors were omitted from analysis (n = 8). Thus, 36 studies remained in the data analysis.

### Reference intervals for ICP and LCSF_op_

**Fig. 2:**
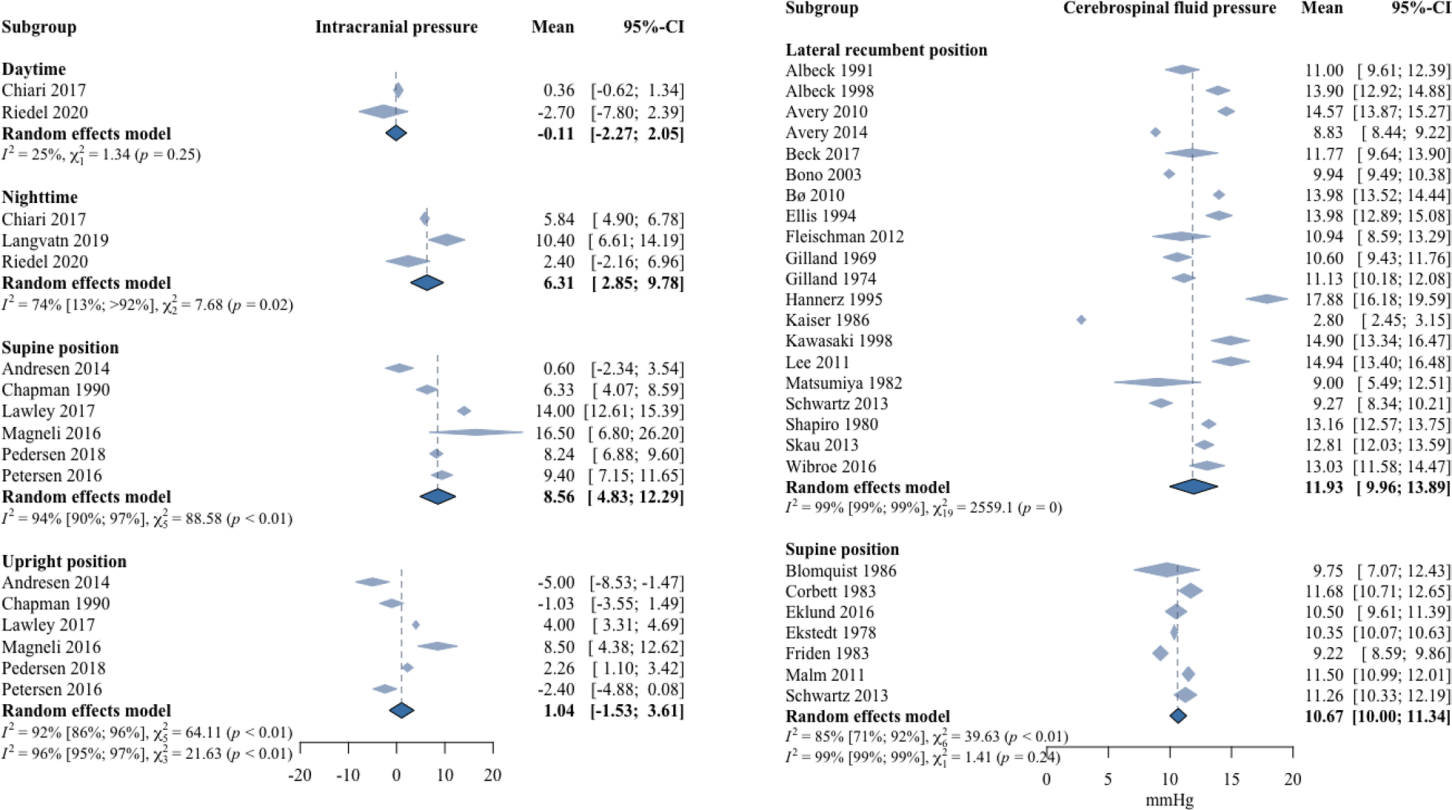
Forest plot of lumbar cerebrospinal fluid pressure and intracranial pressure. Two forest plots describing the weighted average of lumbar cerebrospinal fluid pressure (LCSF_op_) and intracranial pressure (ICP) reported in each included study. The LCSF_op_ is presented to the left and grouped into different body positions: lateral recumbent position and supine position. ICP is presented to the right, and also sub-grouped into different body positions: daytime, nighttime, supine and upright position

Reference intervals differed significantly between body positions (e.g., lateral recumbent position LCSF_op_ versus supine LCSF_op_ (P = 0.04), and upright ICP versus supine ICP (P < 0.01)). Details are shown in Table 2 for both ICP and LCSF_op_. Nine studies provided values for ICP measured intracranially. Reference intervals were subdivided into four groups based on body position: (1) supine position with a mean ICP of 8.6 mmHg (SD 4.7, reference interval 0.9 to 16.3 mmHg), (2) upright position with a mean ICP of 1.0 mmHg (SD 4.3, reference interval − 5.9 to 8.3 mmHg), (3) continuous daytime measurement with a mean ICP of − 0.1 mmHg (SD 7.4, reference interval − 12.0 to 12.2 mmHg), and (4) continuous nighttime measurement with a mean ICP of 6.3 mmHg (SD 13.3, reference interval − 15.8 to 28.2) (Fig. 2, Table 2).

**Table 2:** Reference vales for intracranial pressure and lumbar cerebrospinal fluid pressure in different body positions

	No. of studies	No. of participants	Mean (reference interval) [mmHg]	Mean (reference interval) [cmH_2_O]
ICP				
Supine	6	62	8.6 (0.9–16.3)	11.7 (1.2–22.2)
Upright	6	62	1.0 (– 5.9 to 8.3)	1.3 (− 8.7–11.2)
Daytime	2	45	− 0.1 (− 12.0–12.2)	− 0.15 (− 16.3–16.6)
Nighttime	3	57	6.3 (-15.8–28.2)	8.6 (-21.5–38.3)
CSF_op_				
Supine	7	389	10.7 (5.7–15.5)	14.4 (7.5–21.1)
Lateral recumbent	21	13,359	11.9 (7.2–16.8)	16.3 (9.8–22.8)

Twenty-seven studies provided values for LCSF_op_ measured by lumbar puncture. LCSF_op_ was subdivided into two groups: (1) the supine position with a mean LCSF_op_ of 10.7 mmHg (SD 3.0, reference interval 5.7 to 15.5 mmHg), and (2) the lateral recumbent position with a mean LCSF_op_ of 11.9 mmHg (SD 2.9, reference interval 7.2 to 16.8 mmHg) (Fig. 2, Table 2). There was a statistical difference between ICP values in the supine position and LCSF_op_ values in the supine position (P = 0.03). There was no statistical difference between reference intervals for LCSF_op_ based solely on the healthy population compared to reference intervals for LCSF_op_ based on the pseudo-healthy population. The difference between healthy individuals and pseudo-healthy individuals in lateral recumbent position was 1.4 mmHg (P = 0.34) and in supine position 1.0 mmHg (P = 0.73).

### Quality of included studies

The overall methodological quality of the study designs was moderate, see Additional file 3: Appendix 3. Out of seven, the average score of the included studies was 4.5. None of the studies in which ICP was measured were based on completely healthy individuals, and in only two studies, the primary aim was to determine reference intervals for ICP. For the studies in which ICP was measured, the overall methodological study quality was moderate (average study quality = 4/7). In the group of LCSF_op_, the overall study quality was generally higher (average study quality = 5/7) and a few studies included completely healthy individuals (n = 13) [5, 6, 23, 27–35]. In general, studies with LCSF_op_ consisted of a significantly higher number of study participants (Table 1).

## Discussion

This systematic review aimed to establish reference intervals for normal ICP and LCSF_op_. Through an extensive literature search, 44 studies examining either ICP or LCSF_op_ in pseudo-healthy or normal individuals were identified, and of these, 36 provided sufficient pressure values and statistical data to calculate reference intervals. Nine studies reported ICP values, while 27 reported LCSF_op_ values. The overall study quality was moderate. For ICP, the study material was scarce, and the reference intervals were based on small sample sizes. Data on LCSF_op_ were, in comparison, comprehensive in both lateral recumbent position and supine position. Finally, the reference intervals for both ICP and LCSF_op_ were found to differ significantly between body positions, demonstrating the need for position-dependent reference intervals. After completion of the the present study, Bø et al. reported a mean CSF_op_ of 17.5 cmH_2_O (12.9 mmHg) ranging for 4–30 cmH_2_O (2.9–22.1 mmHg) in an prospecitvely sampled out-patient population. These results further supports our conclusions [36].

The literature further indicates that age, weight, neck position, zero point for measurement-site, and monitoring equipment are of great importance in measuring ICP and LCSF_op_ [9, 23, 37–42]. Especially zero point is of uttermost importance when measuring ICP in upright position. In supine and lateral recumbent position, zero-point matters to the extent that it has to be in the patient’s midline, which is a clinical standard in most neurological and neurosurgical departments. However, only nine of the included studies described the zero point of measurements. In terms of the studies reporting on measurement in upright position only two out of six studies reported on zero point. Thus, for the purpose in this systematic review, this was too few, and the reference values were not corrected for zero point.

Although included studies did not provide detailed information regarding age, gender and weight and their influence on ICP, these factors’ significance has previously been investigated. Pedersen et al. found ICP to be inversely related to age with an average decrease of 0.69 mmHg per decade [39]. For LCSF_op_, one study found a steadily decline after 50 years, that females generally had lower LCSF_op_, and that a high body mass index (BMI) was associated with higher values of LCSF_op_.[41] Hannerz et al. and Whiteley et al. among many other studies, likewise, reported that ICP or LCSF_op_ was higher in obese patients [9, 32, 43–45]. However, the clinical significance of this correlation has been subject to some debate [9, 43].

### Reference intervals for ICP

Due to the still invasive nature of obtaining ICP values, it is ethically unacceptable to examine ICP in healthy normal individuals. Several non-invasive ICP measuring methods have been developed, and though they can be useful to estimate interpatient ICP differences, e.g., during follow-up, none are currently accurate enough to measure absolute ICP values [46, 47]. The studies in this review included pseudo-healthy patients [20, 48–50], and we identified no published studies of ICP on completely healthy individuals. A new telemetric monitoring technique has become available in the last decade, allowing for ICP measurements for several months after implantation [51–53]. This has resulted in ICP measurements in groups outside of the traditional patient categories with suspected ICP disorders. Andresen et al. implanted a telemetric ICP monitor in patients who had a small demarcated intracranial tumor surgically removed, thus establishing a pseudo-healthy cohort [48]. Though this cohort is not completely healthy, long-term measurements via the telemetric ICP monitor provide ICP values at a time when intracranial conditions, including ICP, can be considered normal. This is an improvement, since previous research is mainly based on patients undergoing diagnostic evaluation for a suspected ICP disorder [20, 39, 54, 55]. Future research to establish normal ICP, until quantitative non-invasive technology is developed, could benefit from this telemetric monitoring technique and, hopefully, expand the material on ICP values obtained in pseudo-healthy humans. However, there are considerable issues concerning spontaneous drift in baseline pressures with the telemetric technique. Drift may occur at any time after implantation. In a previous study, we reported the median drift to be 2.5 mmHg after a median implantation time of 241 days. This study, furthermore, suggested that especially reimplantation of a new sensor into the burr hole of a previous telemetric ICP sensor might be related to measurement errors including drift in baseline pressures [51]. Thus, in future studies on the subject, we recommend (1) a new burr hole for the telemetric ICP sensor, and (2) that the telemetric sensors are tested for zero drift after explantation.

Telemetric ICP monitoring requires implantation of a telemetric ICP sensor. The implantation is comparable to implantation of a cable-based ICP sensor. It does, however, limits the need for repeated invasive procedures (e.g. repeated ICP measurements with a cable-based ICP sensor). In future research of normal ICP, the challenge will thus be to investigate ICP in patients who are as normal as possible in terms of ICP dynamics but still require neurosurgical intervention. A possible cohort could be patients undergoing neurosurgery with removal of a benign intracranial tumor or with clipping of an unruptured aneurysm. Another cohort could be patients with a severe head trauma, who after a full recovery, can be considered without damage to CSF pathways and ICP dynamics [52, 53, 56].

In this review, we found a reference interval for ICP in the supine position from 0.9 to 16.3 mmHg, based on six studies with a total of 62 participants [20, 48, 54, 57–59]. The reference intervals are based on a small sample size, and do, therefore, not represent a clinically-applicable reference interval. Since most humans, and in terms of patients specifically patients with idiopathic intracranial hypertension and normal pressure hydrocephalus, spend the majority of their lives in the upright (vertical) position, a reference interval for this specific position is needed for interpreting diagnostic long-term ICP monitoring. Based on six studies with a total of 62 participants [20, 39, 48, 57–59], this review found a reference intervals for ICP in the upright position from − 6.2 to 8.0 mmHg. Though too wide to serve as a clinically-applicable reference interval, the data show that negative ICP values can be normal in an upright position [48, 60].

For daytime ICP, the established reference intervals were likewise based on a limited number of studies (n = 2) and study participants (n = 45) [42, 61]. Furthermore, there are large interpersonal differences in the amount of time spend in an upright position during the day. These factors probably result in the wide reference interval from − 12.0 to 12.2 mmHg. As with the reference interval for ICP in the upright position, this does not serve as a practical tool in clinical decision making. For night-time, we were able to establish a reference interval from -15.8 to 28.2 mmHg. Besides a limited number of studies (n = 3) and participants (n = 57) addressing the matter [42, 61, 62], body position during sleep may vary considerably among patients. Furthermore, the degree of sleep apnoea has also been found to highly impact ICP [42]. Combined, this could potentially cause significant variations in measured ICP and, result in the very wide and not clinically useful reference interval. Unfortunately, the included studies did not provide information on body position during sleep or sleep apnoea coherent to ICP values.

There probably is considerable interpersonal differences in normal ICP. Thus, what will be normal in a 10 year-old normal weight girl may differ from an 80 year-old obese man. Therefore, the idea of using a single value of ICP as a way to distinguish normal values from pathological values, is possibly not biologically natural. Since ICP is highly dependent on body position and activities, one needs to consider if the values are achieved from bed-bound or active patients. Included data is currently to scarce to make a model accounting for these factors. Instead, we suggest a standardized controlled setting defining “baseline ICP” in different body positions related to patient characteristics (such as age, gender and weight), e.g. 10 min measurement following 20 min supine position.

### Reference intervals for LCSF_op_

The majority of included studies on LCSF_op_ (81%) performed pressure measurement in a lateral recumbent position [7, 9, 22, 27, 28, 30–35, 41, 43, 63–73], and a reference interval in this position from 7.2 to 16.8 mmHg was established. There was no significant difference between the reference intervals in healthy individuals versus pseudo-healthy individuals. The established reference interval is similar to the reference of 7 to 15 mmHg routinely used in clinical practice [5, 17, 22, 23, 48]. Seven studies obtained LCSF_op_ values in supine position [5, 7, 23, 29, 63, 74, 75], resulted in a reference interval from 5.7 to 15.5 mmHg. Participants in three out of the seven studies were completely healthy [5, 23, 29]. There was no significant difference between reference intervals in healthy individuals versus pseudo-healthy individuals.When comparing supine LCSF_op_ to supine ICP, we found a difference between the means corresponding to 2.1 mmHg (P = 0.03). These results suggest that supine LCSF_op_ is not optimal as a surrogate marker of supine ICP. To our knowledge, there are no studies that compare simultaneously measured supine ICP with supine LCSF_op_.

### Posture-dependent pressure differences

Postural-dependent pressure changes have mainly been evaluated in studies investigating ICP [18, 58]. Andresen et al. compared lumbar recumbent position to supine position in 31 patients with intracranial ICP monitoring. They found that ICP increased approximately 5 mmHg in the lateral recumbent position, a significantly higher difference than found between LCSF_op_ in recumbent position versus supine position in this review [18]. This postural-related difference in ICP may be caused by spine flexion and, in particular, flexion of the neck during lateral recumbent position in the study by Andresen et al. [18]. A flexed neck could theoretically compress the jugular veins, thus hindering the venous return to the heart from the head, thereby increasing ICP [40, 76–78]. Many studies have emphasized the importance of a neutral neck during the measurement of LCSF_op_ [22, 23, 40, 76]. Though statistically significant, the mean LCSF_op_ difference of 1.2 mmHg between the lateral recumbent position and supine position hardly has any clinical implications (Fig. 2). This relatively small difference might be explained by LCSF_op_ in the lateral recumbent position having been measured with the neck in a neutral position. Unfortunately, the significant change in LCSF_op_ caused by neck position alone has not previously been subject to much debate. Thus, only six of the included studies examining LCSF_op_ documented that LCSF_op_ was obtained simultaneously with a neutral neck position [6, 23, 27, 30, 33, 43]. To establish an accurate reference interval of LCSF_op,_ the neck should be held in a neutral position during all LCSF_op_ measurements.

## Conclusions

In this systematic review, we aimed to establish reference intervals for ICP and LCSF_op_.

The data on ICP was not sufficient to establish clinically applicable reference intervals for either supine position, upright position, daytime, or night-time. Negative ICP in upright position do however seem to be normal. For LCSF_op_ we estimated clinically-applicable reference intervals in both lateral recumbent position (6.3 to 15.9 mmHg) and supine position (5.3 to 15.1 mmHg). This systematic review highlights the need for future research within the field of reference intervals for ICP measures.

## Supplementary Information

**Additional file 1: Appendix 1.** PRISMA checklist.

**Additional file 2: Appendix 2.** Search strategy.

**Additional file 3: Appendix 3.** Quality assessment.

## Data Availability

The data set used and analysed during the current study is available from the corresponding author on reasonable request.
