# Supplementary material for: Reference values for intracranial pressure and lumbar cerebrospinal fluid pressure: a systematic review
Source: Fluids Barriers CNS. 2021 Apr 13;18:19. doi: 10.1186/s12987-021-00253-4 (PMC8045192; doi:10.1186/s12987-021-00253-4)
Supplement: Supplementary file 2 — Additional file 2: Appendix 2. Search strategy. [file 12987_2021_253_MOESM2_ESM.docx]

| Search | Add to builder | Query | Items found | Time |
| --- | --- | --- | --- | --- |
| [#11](https://www.ncbi.nlm.nih.gov/pubmed) | [Add](https://www.ncbi.nlm.nih.gov/pubmed) | Search **(((reference values[MeSH Terms]) OR ((((reference value*) OR reference range*) OR normal range*) OR normal value*))) AND ((intracranial pressure[MeSH Terms]) OR (((intracranial pressure*) OR subarachnoid pressure*) OR intracerebral pressure*))** | [468](https://www.ncbi.nlm.nih.gov/pubmed/?cmd=HistorySearch&querykey=11) | 07:57:33 |
| [#10](https://www.ncbi.nlm.nih.gov/pubmed) | [Add](https://www.ncbi.nlm.nih.gov/pubmed) | Search **(intracranial pressure[MeSH Terms]) OR (((intracranial pressure*) OR subarachnoid pressure*) OR intracerebral pressure*)** | [24850](https://www.ncbi.nlm.nih.gov/pubmed/?cmd=HistorySearch&querykey=10) | 07:57:22 |
| [#9](https://www.ncbi.nlm.nih.gov/pubmed) | [Add](https://www.ncbi.nlm.nih.gov/pubmed) | Search **((intracranial pressure*) OR subarachnoid pressure*) OR intracerebral pressure*** | [24850](https://www.ncbi.nlm.nih.gov/pubmed/?cmd=HistorySearch&querykey=9) | 07:57:09 |
| [#8](https://www.ncbi.nlm.nih.gov/pubmed) | [Add](https://www.ncbi.nlm.nih.gov/pubmed) | Search **((intracranial pressure*) AND subarachnoid pressure*) AND intracerebra åressure*** | [0](https://www.ncbi.nlm.nih.gov/pubmed/?cmd=HistorySearch&querykey=8) | 07:56:29 |
| [#7](https://www.ncbi.nlm.nih.gov/pubmed) | [Add](https://www.ncbi.nlm.nih.gov/pubmed) | Search **intracranial pressure[MeSH Terms]** | [14674](https://www.ncbi.nlm.nih.gov/pubmed/?cmd=HistorySearch&querykey=7) | 07:55:59 |
| [#4](https://www.ncbi.nlm.nih.gov/pubmed) | [Add](https://www.ncbi.nlm.nih.gov/pubmed) | Search **(reference values[MeSH Terms]) OR ((((reference value*) OR reference range*) OR normal range*) OR normal value*)** | [230774](https://www.ncbi.nlm.nih.gov/pubmed/?cmd=HistorySearch&querykey=4) | 07:55:34 |
| [#3](https://www.ncbi.nlm.nih.gov/pubmed) | [Add](https://www.ncbi.nlm.nih.gov/pubmed) | Search **(((reference value*) OR reference range*) OR normal range*) OR normal value*** | [230774](https://www.ncbi.nlm.nih.gov/pubmed/?cmd=HistorySearch&querykey=3) | 07:55:25 |
| [#2](https://www.ncbi.nlm.nih.gov/pubmed) | [Add](https://www.ncbi.nlm.nih.gov/pubmed) | Search **reference values[MeSH Terms]** | [157289](https://www.ncbi.nlm.nih.gov/pubmed/?cmd=HistorySearch&querykey=2) | 07:54:42 |

**Search strategy**

**Search date: July 16^th^ 2019**

**Pubmed: (468)**

**EMbase (497)**

| \|  \| [# ▲](http://ovidsp.dc1.ovid.com.ep.fjernadgang.kb.dk/sp-3.33.0b/ovidweb.cgi?&S=MFDCFPEENFACCNOLKPCKLGMOPJJIAA00&Sort+Sets=descending) \| **Searches** \| **Results** \| **Type** \| **Actions** \| **Annotations** \| \| --- \| --- \| --- \| --- \| --- \| --- \| --- \| \|  \| \| \| \| \| \| \| \|  \| 1 \| exp reference value/ \| 126777 \| Advanced \| [Display Results](http://ovidsp.dc1.ovid.com.ep.fjernadgang.kb.dk/sp-3.33.0b/ovidweb.cgi?&S=MFDCFPEENFACCNOLKPCKLGMOPJJIAA00&SELECT=S.sh%7c&R=1&Process+Action=display)  [More](http://ovidsp.dc1.ovid.com.ep.fjernadgang.kb.dk/sp-3.33.0b/ovidweb.cgi) \|  \| \|  \| 2 \| exp intracranial pressure/ \| 20943 \| Advanced \| [Display Results](http://ovidsp.dc1.ovid.com.ep.fjernadgang.kb.dk/sp-3.33.0b/ovidweb.cgi?&S=MFDCFPEENFACCNOLKPCKLGMOPJJIAA00&SELECT=S.sh%7c&R=2&Process+Action=display)  [More](http://ovidsp.dc1.ovid.com.ep.fjernadgang.kb.dk/sp-3.33.0b/ovidweb.cgi) \|  \| \|  \| 3 \| (reference value* or reference interval* or reference range* or normal range* or normal value*).af. \| 229896 \| Advanced \| [Display Results](http://ovidsp.dc1.ovid.com.ep.fjernadgang.kb.dk/sp-3.33.0b/ovidweb.cgi?&S=MFDCFPEENFACCNOLKPCKLGMOPJJIAA00&SELECT=S.sh%7c&R=3&Process+Action=display)  [More](http://ovidsp.dc1.ovid.com.ep.fjernadgang.kb.dk/sp-3.33.0b/ovidweb.cgi) \|  \| \|  \| 4 \| 1 or 3 \| 230831 \| Advanced \| [Display Results](http://ovidsp.dc1.ovid.com.ep.fjernadgang.kb.dk/sp-3.33.0b/ovidweb.cgi?&S=MFDCFPEENFACCNOLKPCKLGMOPJJIAA00&SELECT=S.sh%7c&R=4&Process+Action=display)  [More](http://ovidsp.dc1.ovid.com.ep.fjernadgang.kb.dk/sp-3.33.0b/ovidweb.cgi) \|  \| \|  \| 5 \| (intracranial pressure* or subarachnoid pressure* or intracerebral pressure* or brain pressure*).af. \| 32602 \| Advanced \| [Display Results](http://ovidsp.dc1.ovid.com.ep.fjernadgang.kb.dk/sp-3.33.0b/ovidweb.cgi?&S=MFDCFPEENFACCNOLKPCKLGMOPJJIAA00&SELECT=S.sh%7c&R=5&Process+Action=display)  [More](http://ovidsp.dc1.ovid.com.ep.fjernadgang.kb.dk/sp-3.33.0b/ovidweb.cgi) \|  \| \|  \| 6 \| 2 or 5 \| 32602 \| Advanced \| [Display Results](http://ovidsp.dc1.ovid.com.ep.fjernadgang.kb.dk/sp-3.33.0b/ovidweb.cgi?&S=MFDCFPEENFACCNOLKPCKLGMOPJJIAA00&SELECT=S.sh%7c&R=6&Process+Action=display)  [More](http://ovidsp.dc1.ovid.com.ep.fjernadgang.kb.dk/sp-3.33.0b/ovidweb.cgi) \|  \| \|  \| 7 \| 4 and 6 \| 497 \| Advanced \| [Display Results](http://ovidsp.dc1.ovid.com.ep.fjernadgang.kb.dk/sp-3.33.0b/ovidweb.cgi?&S=MFDCFPEENFACCNOLKPCKLGMOPJJIAA00&SELECT=S.sh%7c&R=7&Process+Action=display)  [More](http://ovidsp.dc1.ovid.com.ep.fjernadgang.kb.dk/sp-3.33.0b/ovidweb.cgi) \|  \| |
| --- | --- | --- | --- | --- | --- | --- | --- | --- | --- | --- | --- | --- | --- | --- | --- | --- | --- | --- | --- | --- | --- | --- | --- | --- | --- | --- | --- | --- | --- | --- | --- | --- | --- | --- | --- | --- | --- | --- | --- | --- | --- | --- | --- | --- | --- | --- | --- | --- | --- | --- | --- | --- | --- | --- | --- | --- | --- | --- | --- | --- | --- | --- | --- |

**Cochrane (229)**

ID Search Hits

#1 MeSH descriptor: [Reference Values] explode all trees 9328

#2 MeSH descriptor: [Intracranial Pressure] explode all trees 333

#3 intracranial pressure 2258

#4 reference value 10501

#5 #1 OR #4 18974

#6 #2 OR #3 2258

#7 #5 AND #6 229

**Web of sciences (1316)**

| Search History | | | | | |
| --- | --- | --- | --- | --- | --- |
| **Set** | **Results** | **Save History / Create AlertOpen Saved History** | **Edit Sets** | **Combine Sets**  **AND   OR**  **Combine** | **Delete Sets**  **Select All  Delete** |
|  | | | | | |
| # 5 | [**1,316**](http://apps.webofknowledge.com.ep.fjernadgang.kb.dk/summary.do?product=WOS&doc=1&qid=5&SID=E2Vytk5IDVvcoEuZSZU&search_mode=CombineSearches&update_back2search_link_param=yes) | #4 AND #3  *Indexes=SCI-EXPANDED, SSCI, A&HCI, CPCI-S, CPCI-SSH, BKCI-S, BKCI-SSH, ESCI, CCR-EXPANDED, IC Timespan=All years* | [Edit](http://apps.webofknowledge.com.ep.fjernadgang.kb.dk/WOS_AdvancedSearch_input.do?product=WOS&SID=E2Vytk5IDVvcoEuZSZU&search_mode=AdvancedSearch&replaceSetId=5&editState=init) |  |  |
|  | | | | | |
| # 4 | [**26,507**](http://apps.webofknowledge.com.ep.fjernadgang.kb.dk/summary.do?product=WOS&doc=1&qid=4&SID=E2Vytk5IDVvcoEuZSZU&search_mode=AdvancedSearch&update_back2search_link_param=yes) | TS=(intracranial pressure OR subarachnoid pressure OR intracerebral pressure)  *Indexes=SCI-EXPANDED, SSCI, A&HCI, CPCI-S, CPCI-SSH, BKCI-S, BKCI-SSH, ESCI, CCR-EXPANDED, IC Timespan=All years* | [Edit](http://apps.webofknowledge.com.ep.fjernadgang.kb.dk/WOS_AdvancedSearch_input.do?product=WOS&SID=E2Vytk5IDVvcoEuZSZU&search_mode=AdvancedSearch&replaceSetId=4&editState=init) |  |  |
|  | | | | | |
| # 3 | [**554,501**](http://apps.webofknowledge.com.ep.fjernadgang.kb.dk/summary.do?product=WOS&doc=1&qid=3&SID=E2Vytk5IDVvcoEuZSZU&search_mode=AdvancedSearch&update_back2search_link_param=yes) | TS=(reference value* OR reference range* OR normal range* OR normal value*)  *Indexes=SCI-EXPANDED, SSCI, A&HCI, CPCI-S, CPCI-SSH, BKCI-S, BKCI-SSH, ESCI, CCR-EXPANDED, IC Timespan=All years* | [Edit](http://apps.webofknowledge.com.ep.fjernadgang.kb.dk/WOS_AdvancedSearch_input.do?product=WOS&SID=E2Vytk5IDVvcoEuZSZU&search_mode=AdvancedSearch&replaceSetId=3&editState=init) |  |  |
|  | | | | | |
| # 2 | [**156,951**](http://apps.webofknowledge.com.ep.fjernadgang.kb.dk/summary.do?product=WOS&doc=1&qid=2&SID=E2Vytk5IDVvcoEuZSZU&search_mode=AdvancedSearch&update_back2search_link_param=yes) | TS=(Reference value*)  *Indexes=SCI-EXPANDED, SSCI, A&HCI, CPCI-S, CPCI-SSH, BKCI-S, BKCI-SSH, ESCI, CCR-EXPANDED, IC Timespan=All years* | [Edit](http://apps.webofknowledge.com.ep.fjernadgang.kb.dk/WOS_AdvancedSearch_input.do?product=WOS&SID=E2Vytk5IDVvcoEuZSZU&search_mode=AdvancedSearch&replaceSetId=2&editState=init) |  |  |
|  | | | | | |
| # 1 | [**22,567**](http://apps.webofknowledge.com.ep.fjernadgang.kb.dk/summary.do?product=WOS&doc=1&qid=1&SID=E2Vytk5IDVvcoEuZSZU&search_mode=AdvancedSearch&update_back2search_link_param=yes) | TS=(intracranial pressure*)  *Indexes=SCI-EXPANDED, SSCI, A&HCI, CPCI-S, CPCI-SSH, BKCI-S, BKCI-SSH, ESCI, CCR-EXPANDED, IC Timespan=All years* | [Edit](http://apps.webofknowledge.com.ep.fjernadgang.kb.dk/WOS_AdvancedSearch_input.do?product=WOS&SID=E2Vytk5IDVvcoEuZSZU&search_mode=AdvancedSearch&replaceSetId=1&editState=init) |  |  |
|  | | | | | |

**Manual search of the reference list of included studies**

6 studies identified

Total number identified: 2516

Duplicates removed: 725

Number in list: 1791
