# Supplementary material for: Reference values for intracranial pressure and lumbar cerebrospinal fluid pressure: a systematic review
Source: Fluids Barriers CNS. 2021 Apr 13;18:19. doi: 10.1186/s12987-021-00253-4 (PMC8045192; doi:10.1186/s12987-021-00253-4)
Supplement: Supplementary file 3 — Additional file 3: Appendix 3. Quality assessment. [file 12987_2021_253_MOESM3_ESM.docx]

|  | Were the criteria for inclusion in the sample clearly defined? | Were the study subjects and the settings described in detail? | Was the measurement method described in detail?* | Were confounding factors identified? | Were strategies to deal with confounding factors stated? | Was the included population completely healthy?* | Was appropriate statistical analysis used? | Score |
| --- | --- | --- | --- | --- | --- | --- | --- | --- |
| *Albeck 1991* | 1 | 1 | 1 | 1 | 1 | 1 | 1 | 7 |
| *Albeck 1998* | 1 | 1 | 1 | 1 | 1 | 1 | 1 | 7 |
| *Andresen 2014* | 1 | 1 | 1 | 1 | 1 | 0 | 1 | 6 |
| *Avery 2010* | 1 | 1 | 0 | 0 | 0 | 1 | 1 | 4 |
| *Avery 2014* | 0 | 0 | 0 | 0 | 0 | 0 | 1 | 1 |
| *Beck 2017* | 1 | 1 | 1 | 1 | 1 | 0 | 1 | 6 |
| *Blomquist 1986* | 1 | 1 | 1 | 1 | 0 | 0 | 0 | 4 |
| *Bono 2003* | 1 | 1 | 0 | 0 | 0 | 0 | 1 | 3 |
| *Bø 2010* | 1 | 1 | 1 | 0 | 0 | 0 | 1 | 4 |
| *Chapman 1990* | 0 | 1 | 1 | 1 | 1 | 0 | 0 | 4 |
| *Chiari 2017* | 1 | 1 | 1 | 0 | 0 | 0 | 1 | 4 |
| *Corbett 1983* | 1 | 1 | 1 | 1 | 0 | 1 | 1 | 6 |
| *Eklund 2016* | 1 | 1 | 1 | 1 | 1 | 1 | 1 | 7 |
| *Ekstedt 1978* | 1 | 1 | 1 | 1 | 1 | 0 | 1 | 6 |
| *Ellis 1994* | 0 | 1 | 1 | 0 | 0 | 0 | 1 | 3 |
| *Fleischman 2012* | 1 | 1 | 0 | 0 | 0 | 0 | 1 | 3 |
| *Friden 1983* | 1 | 0 | 1 | 1 | 1 | 0 | 1 | 5 |
| *Gilland 1969* | 1 | 1 | 0 | 0 | 0 | 1 | 1 | 4 |
| *Gilland 1974* | 1 | 1 | 1 | 1 | 1 | 1 | 1 | 7 |
| *Gonzalez 2017* | 1 | 1 | 1 | 1 | 1 | 0 | 1 | 6 |
| *Hannerz 1995* | 1 | 1 | 0 | 0 | 0 | 1 | 1 | 4 |
| *Kaiser 1986* | 1 | 0 | 0 | 0 | 0 | 0 | 1 | 2 |
| *Kawasaki 1998* | 0 | 0 | 0 | 0 | 0 | 0 | 1 | 1 |
| *Lakke 1968* | 0 | 1 | 1 | 1 | 1 | 1 | 0 | 5 |
| *Langvatn 2019* | 1 | 1 | 1 | 1 | 0 | 0 | 1 | 5 |
| *Lawley 2017* | 1 | 1 | 1 | 1 | 1 | 0 | 1 | 6 |
| *Lee 2011* | 1 | 1 | 1 | 1 | 1 | **0** | 1 | 6 |
| *Lundberg 1960* | 0 | 1 | 1 | 0 | 0 | 0 | 0 | 2 |
| *Magneli 2016* | 0 | 1 | 1 | 1 | 1 | 0 | 0 | 4 |
| *Mahr 2016* | 1 | 1 | 1 | 0 | 0 | 0 | 0 | 3 |
| *Malm 2011* | 1 | 1 | 1 | 1 | 0 | 1 | 1 | 6 |
| *Martin 1978* | 0 | 1 | 1 | 0 | 0 | 1 | 0 | 3 |
| *Matsumiya 1982* | 1 | 1 | 1 | 0 | 0 | 0 | 1 | 4 |
| *Pedersen 2018* | 1 | 1 | 1 | 1 | 0 | 0 | 1 | 5 |
| *Petersen 2016* | 1 | 1 | 1 | 1 | 1 | 0 | 1 | 6 |
| *Purvin 2000* | 0 | 1 | 0 | 0 | 0 | 0 | 1 | 2 |
| *Riedel 2020* | 1 | 1 | 1 | 1 | 1 | 0 | 1 | 6 |
| *Schwartz 2013* | 1 | 1 | 1 | 1 | 0 | 1 | 1 | 6 |
| *Shapiro 1980* | 0 | 0 | 1 | 1 | 1 | 1 | 1 | 5 |
| *Skau 2013* | 1 | 1 | 1 | 1 | 0 | 1 | 1 | 6 |
| *Skipper 2019* | 1 | 1 | 0 | 0 | 0 | 1 | 1 | 4 |
| *Sugita 1985* | 0 | 1 | 1 | 0 | 0 | 0 | 0 | 2 |
| *Whiteley 2006* | 1 | 1 | 1 | 1 | 0 | 0 | 1 | 5 |
| *Wibroe 2016* | 1 | 1 | 1 | 0 | 0 | 0 | 1 | 4 |

*1) Was the exposure measured in a valid and reliable way?*

*2) Were the outcomes measured in a valid and reliable way?*

*The Joanna Briggs Institute battery of quality appraisal tools amended version*
